# Association Between Endotype of Prematurity and Cystic Periventricular Leukomalacia: A Bayesian Model-Averaged Meta-Analysis

**DOI:** 10.3390/children12081065

**Published:** 2025-08-13

**Authors:** Neirude P. A. Lissone, Tamara M. Hundscheid, Gloria M. Galán-Henríquez, Gema E. González-Luis, František Bartoš, Eduardo Villamor

**Affiliations:** 1 Division of Neonatology, MosaKids Children’s Hospital, Maastricht University Medical Center (MUMC+), Research Institute for Oncology and Reproduction (GROW), Maastricht University, 6202 AZ Maastricht, The Netherlands; 2 Department of Pediatrics, Hospital Universitario Materno-Infantil de Canarias, E-35016 Las Palmas de Gran Canaria, Spain; 3 Department of Psychology, University of Amsterdam, 1105 AZ Amsterdam, The Netherlands

**Keywords:** preterm birth, chorioamnionitis, hypertensive disorders of pregnancy, growth restriction, periventricular leukomalacia

## Abstract

**Introduction:** Pathophysiological pathways—or endotypes—leading to prematurity can be clustered into two groups: infection/inflammation and dysfunctional placentation. We aimed to perform a systematic review and meta-analysis of studies exploring the association between these endotypes and cystic periventricular leukomalacia (cPVL). **Methods:** PubMed and Embase were searched for observational studies examining preterm infants and reporting data on the association between endotype of prematurity and cPVL. Chorioamnionitis represented the infectious–inflammatory endotype, while dysfunctional placentation proxies were hypertensive disorders of pregnancy (HDPs) and small for gestational age (SGA)/intrauterine growth restriction (IUGR). Bayesian model-averaged (BMA) meta-analysis was used to calculate Bayes factors (BFs). The BF_10_ is the ratio of the probability of the data under the alternative hypothesis (H_1;_ presence of association) over the probability of the data under the null hypothesis (H_0;_ absence of association). **Results:** Of 1141 potentially relevant studies; 67 (108,571 infants) were included. The BMA analysis showed strong evidence in favor of a positive association between chorioamnionitis and cPVL (OR 1.58; 95% CrI 1.12 to 2.20; BF_10_ = 20.5) and extreme evidence in favor of a negative association between HDPs and cPVL (OR 0.63; 95% CrI 0.54 to 0.75; BF_10_ = 2937). The evidence for the SGA/IUGR group was inconclusive (OR 0.87; 95% CrI 0.75 to 1.01; BF_10_ = 1.41). **Conclusions:** This Bayesian meta-analysis provides evidence indicative of an association between antenatal infection–inflammation and an increased risk of developing cPVL in preterm infants. Conversely, infants exposed to HDPs are less likely to develop cPVL.

## 1. Introduction

Periventricular leukomalacia (PVL) is one of the leading causes of long-term neurodevelopmental impairment in preterm infants [1,2,3,4,5]. The pathogenesis of PVL is a complex process that is dependent on the interaction of various maturation-related factors, which collectively render preterm infants particularly susceptible to the development of cerebral white matter injury. The two principal initiating pathogenic factors in PVL appear to be cerebral ischemia and antenatal or neonatal infection/inflammation [1,2,3,4]. These two upstream mechanisms activate two critical downstream mechanisms: excitotoxicity and free radical attack by reactive oxygen and nitrogen species, which ultimately result in the death of the vulnerable white matter [1,2,3,4]. PVL occurs in two overlapping forms: cystic PVL (cPVL), in which the focal necrosis of the white mater is macroscopic and evolves into multiple cysts, and non-cystic PVL, in which the focal necrosis is microscopic and evolves into glial scars [1,2,3,4]. To some extent, these variants correlate with distinct clinical outcomes. While cPVL is typically associated with spastic bilateral cerebral palsy, the predominant deficits following diffuse non-cystic white matter injury are cognitive impairment and behavioral, attention, or socialization problems [5].

Although low gestational age (GA) is a major risk factor for the development of PVL, the clinical course of the preterm infant also appears to be of great relevance in predicting the risk of brain injury and abnormal neurodevelopment [4,6]. Furthermore, it is possible that the pathophysiological cascade leading to PVL may be initiated before birth [4,6]. Preterm birth is the result of a pathological process, which may not only contribute to early delivery but may also adversely impact neonatal outcomes. The pathogenic pathways, or endotypes, that lead to very and extremely preterm birth (GA < 32 weeks) can be clustered into two main groups: (1) intrauterine infection/inflammation and (2) dysfunctional placentation [7,8]. The fetal environment differs greatly between the two endotypes of prematurity. In the case of the infectious endotype, the response is inflammatory with release of cytokines and other inflammatory mediators [7,8]. These mediators are directly toxic to premyelinating oligodendrocytes and promote apoptosis and myelin loss [1,9,10,11]. In the case of the placental dysfunction endotype, there is chronic hypoxia and imbalance of angiogenic mediators [7,8]. This induces metabolic and oxidative injury and increases fetal susceptibility to hypoxia–ischemia [12,13,14,15,16,17].

In recent years, our group has analyzed the association of the two endotypes of prematurity with mortality and short-term complications, including bronchopulmonary dysplasia (BPD), intraventricular hemorrhage (IVH), retinopathy of prematurity (ROP), late-onset sepsis (LOS), or patent ductus arteriosus [18,19,20,21,22,23,24,25,26]. We have used chorioamnionitis as a proxy for the infectious–inflammatory endotype and hypertensive disorders of pregnancy (HDPs) and intrauterine growth retardation (IUGR, based on prenatal growth assessment) or being small for GA (SGA, based on birth weight below a given percentile) as proxies for the placental vascular dysfunction endotype [18,19,20,21,22,23,24,25,26]. The objective of the present study was to conduct a systematic review and meta-analysis of the potential association between the endotypes of prematurity and the risk of developing PVL. Our meta-analysis focused on cPVL, the most severe form of PVL, which is widely recognized as a leading cause of major neurodevelopmental impairments, including cerebral palsy, postnatal epilepsy, and cognitive deficits [1,2,3,4].

## 2. Methods

The methodology of this study is based on that of earlier studies by our group on endotypes as risk factors for outcomes of prematurity [18,19,20,26]. The study was performed and reported according to the preferred reporting items for systematic reviews and meta-analyses (PRISMA) and meta-analysis of observational studies in epidemiology (MOOSE) guidelines. The review protocol was registered in PROSPERO database (ID = CRD42020184843). Detailed information on methods is provided as Supplementary Material. The Population, Exposure, Comparison, and Outcome (PECO) question was: Do preterm infants (P) exposed to chorioamnionitis, HDPs, or IUGR (E) have a higher risk of cPVL (O) than preterm infants with no history of exposure (C)?

Since many researchers use small for gestational age (SGA) as a proxy for IUGR, and although the two conditions are not necessarily synonymous [27], we also collected data on SGA. From now on, this group will be referred to as SGA/IUGR.

### 2.1. Sources and Search Strategy

A comprehensive literature search was undertaken using the PubMed and EMBASE from their inception up to February 2024. The search strategy is detailed in the Supplementary Material.

### 2.2. Study Selection

Studies were included if they examined preterm or very low-birth-weight infants (GA < 37 weeks or birth weight < 1500 g) and reported primary data that could be used to measure the association between exposure to CA, HDPs, or SGA/IUGR and cPVL.

### 2.3. Data Extraction, Definitions, and Quality Assessment

Two investigators (NL, TH) independently extracted data from relevant studies using a predetermined data extraction form. In instances where raw data were not reported, unadjusted odds ratios (ORs) were utilized in this meta-analysis. Any definition of cPVL based on either ultrasonography or magnetic resonance imaging (MRI) was accepted. Any definition of chorioamnionitis, HDP, or SGA/IUGR was accepted, but subgroup analysis was performed based on the different definitions (Table 1). When a study used different BW threshold percentiles to define SGA, data from the lowest percentile were included. In addition to chorioamnionitis, the potential association of funisitis with cPVL was also analyzed. As previously described, three different control groups were used for the analysis of funisitis: (1) infants without funisitis (Fun−); (2) infants without funisitis but with chorioamnionitis (Fun−/CA+); and (3) infants who had neither funisitis nor chorioamnionitis (Fun−/CA−) [28]. Methodological quality was assessed using the Newcastle–Ottawa Scale (NOS) for cohort or case–control studies [29].

### 2.4. Bayesian Model-Averaged Meta-Analysis

Effect size of dichotomous variables was expressed as logOR and effect size of continuous variables was expressed using the Hedges’ *g*. Values of logOR and Hedges’ *g* and the corresponding standard error of each individual study were calculated using comprehensive meta-analysis (CMA) V4.0 software (Biostat Inc., Englewood, NJ, USA). The results were further pooled and analyzed by a Bayesian model-averaged (BMA) meta-analysis [30,31]. We performed the BMA in JASP (version 0.95.0), which utilizes the RoBMA R package (version 3.5.1) [32,33]. BMA employs Bayes factors (BFs) and Bayesian model-averaging to evaluate the likelihood of the data under the combination of models assuming the presence vs. the absence of the meta-analytic effect and heterogeneity [30,31]. The BF_10_ is the ratio of the probability of the data under H_1_ over the probability of the data under H_0_. The BF_10_ was interpreted using the evidence categories suggested by Lee & Wagenmakers [34]: <1/100 = extreme evidence for H_0_, from 1/100 to <1/30 = very strong evidence for H_0_, from 1/30 to <1/10 = strong evidence for H_0_, from 1/10 to <1/3 = moderate evidence for H_0_, from 1/3 to <1 weak/inconclusive evidence for H_0_, from 1 to 3 = weak/inconclusive evidence for H_1_, from >3 to 10 = moderate evidence for H_1_, from >10 to 30 = strong evidence for H_1_, from >30 to 100 = very strong evidence for H_1_, and >100 extreme evidence for H_1_. The BF_01_ (ratio of the probability of the data under H_0_ over the probability of the data under H_1_) is the inverse of BF_10_. The BF_rf_ is the ratio of the probability of the data under the random-effects model over the probability of the data under the fixed-effects model.

We used robust Bayesian meta-analysis (RoBMA) to assess the robustness of the results to the potential presence of publication bias, which was expressed as BF_bias_ [35]. The potential moderating effect of the difference in GA (GA of the chorioamnionitis, HDP, or SGA/IUGR group minus GA of the corresponding control group in each individual study) on the association between the condition and cPVL was investigated through Bayesian model-averaged meta-regression (BMA-reg) and expressed as BF_mod_. BMA-reg combines models with different assumptions about the effect, heterogeneity, and moderation, which allows us to examine the evidence for or against each component while accounting for the uncertainty in the remaining components [36]. The categories of strength of the evidence in favor of the random effects (BF_rf_ > 1) or the fixed effect (BF_rf_ < 1), presence vs. absence of publication bias (BF_bias_ >1 vs. BF_bias_ <1), or presence vs. absence of moderation by GA (BF_mod_ > 1 vs. BF_mod_ < 1) were similar to those described above for the BF_10_.

For all the analyses, we used the neonatal-specific empirical prior distributions based on the Cochrane Database of Systematics Reviews [31,37]. Binary outcomes: logOR~Student-t (µ = 0, σ = 0.29, ν = 3), tau (logRR)~Inverse-Gamma (k = 1.80, θ = 0.42); Continuous outcomes: Cohen’s~Student-t (µ = 0, σ = 0.42, ν = 5), tau (Cohen’s d)~Inverse-Gamma (k = 1.68, θ = 0.38) [27,34]. For the meta-regression, we used the prior distributions for the effect size and heterogeneity parameter with the prior distribution on the standardized meta-regression coefficient scaled by 1/2 (i.e., the expected difference in each moderator level corresponding to 1/2 of the mean effect size) [36]. To test the robustness of this analysis, another meta-regression was conducted with the prior distribution on the standardized meta-regression coefficient scaled by 1/4.

## 3. Results

### 3.1. Description of Studies and Risk of Bias Assessment

**Figure 1:**
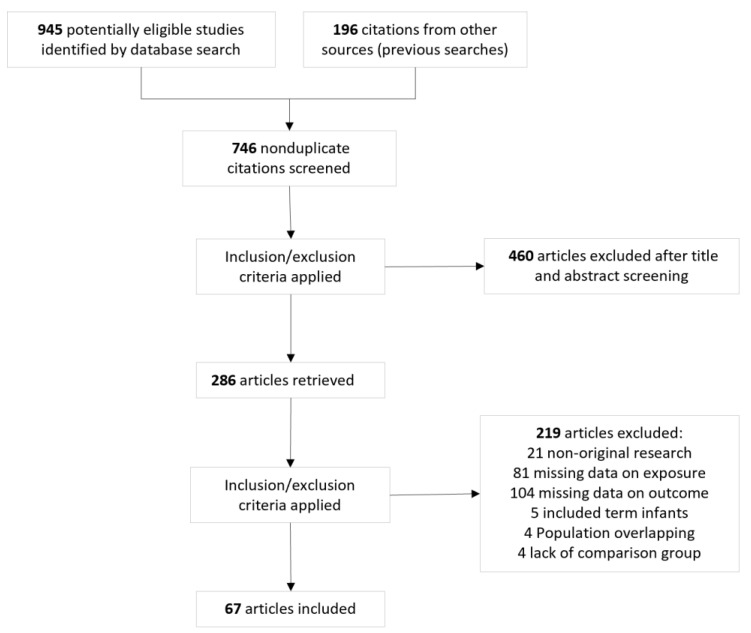
Flow diagram of the systematic search.

The flow diagram of the search process is shown in Figure 1. Of 1141 potentially relevant studies, 67 (108,571 infants) were included [38,39,40,41,42,43,44,45,46,47,48,49,50,51,52,53,54,55,56,57,58,59,60,61,62,63,64,65,66,67,68,69,70,71,72,73,74,75,76,77,78,79,80,81,82,83,84,85,86,87,88,89,90,91,92,93,94,95,96,97,98,99,100,101,102,103,104]. Their characteristics are summarized in Table S1. The quality score of each study according to the NOS is depicted in Table S2. All studies received a score of six or higher, indicating a low to moderate risk of bias.

### 3.2. Bayesian Meta-Analysis

**Figure 2:**
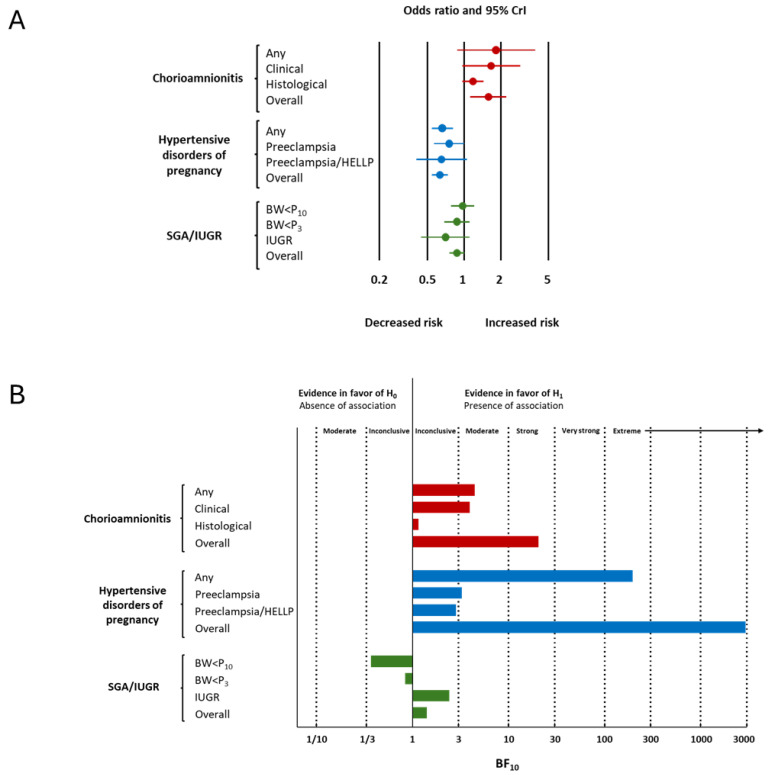
Summary of Bayesian model-averaged (BMA) meta-analyses on the association between endotype of prematurity and cystic periventricular leukomalacia (cPVL). (**A**) Summary of odds ratios (ORs). OR > 1 indicates higher risk of cPVL associated with the condition. (**B**) Summary of Bayes factors (BFs). The BF_10_ is the ratio of the probability of the data under the alternative hypothesis (H_1_) over the probability of the data under the null hypothesis (H_0_). BW, birth weight; CrI, credible interval; IUGR, intrauterine growth restriction; P3, 3rd percentile; P10, 10th percentile; SGA, small for gestational age.

**Figure 3:**
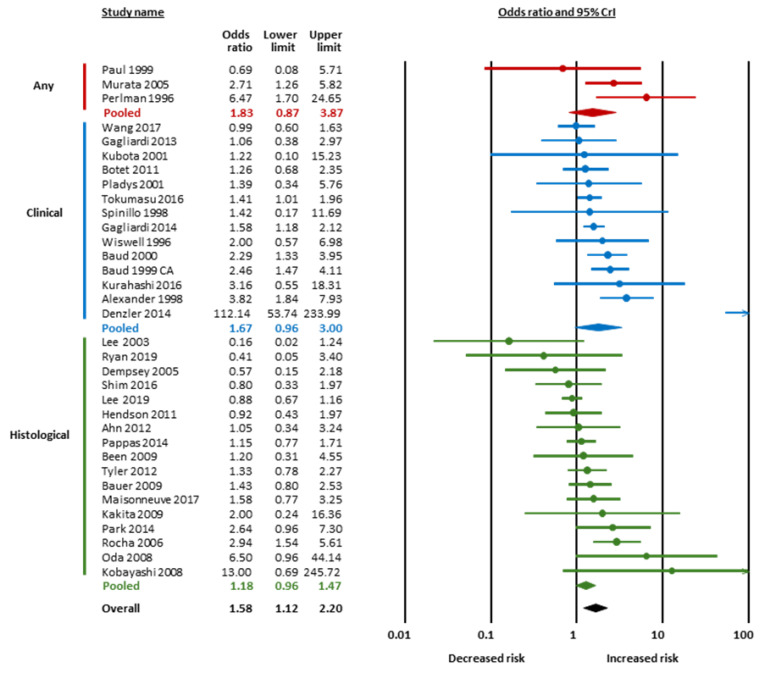
Bayesian model-averaged (BMA) meta-analysis of the association between chorioamnionitis and cystic periventricular leukomalacia. CrI, credible interval [38,39,42,43,44,46,50,51,55,56,58,59,61,64,66,68,70,74,75,76,77,79,80,81,87,88,91,93,95,96,98,100].

**Figure 4:**
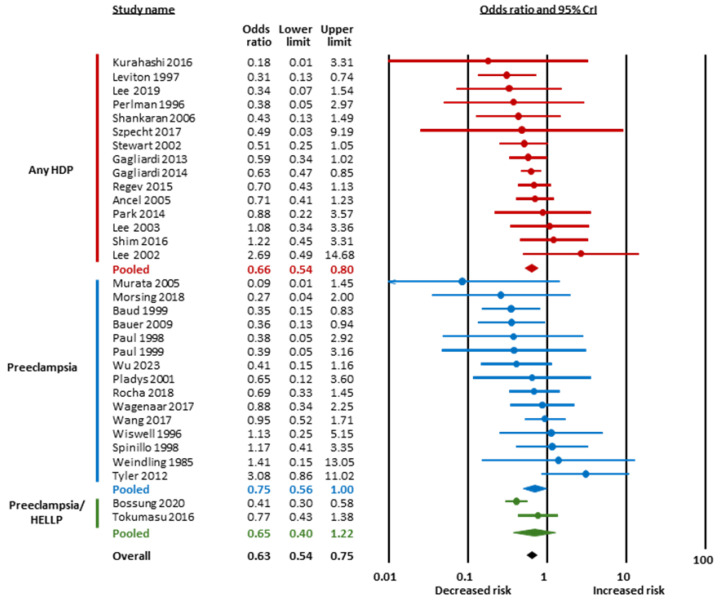
Bayesian model-averaged (BMA) meta-analysis of the association between hypertensive disorders of pregnancy (HDPs) and cystic periventricular leukomalacia. CrI, credible interval [41,42,43,45,55,56,64,67,68,69,73,74,77,78,79,80,81,82,85,90,91,93,94,95,96,97,98,99,100,101,104].

**Figure 5:**
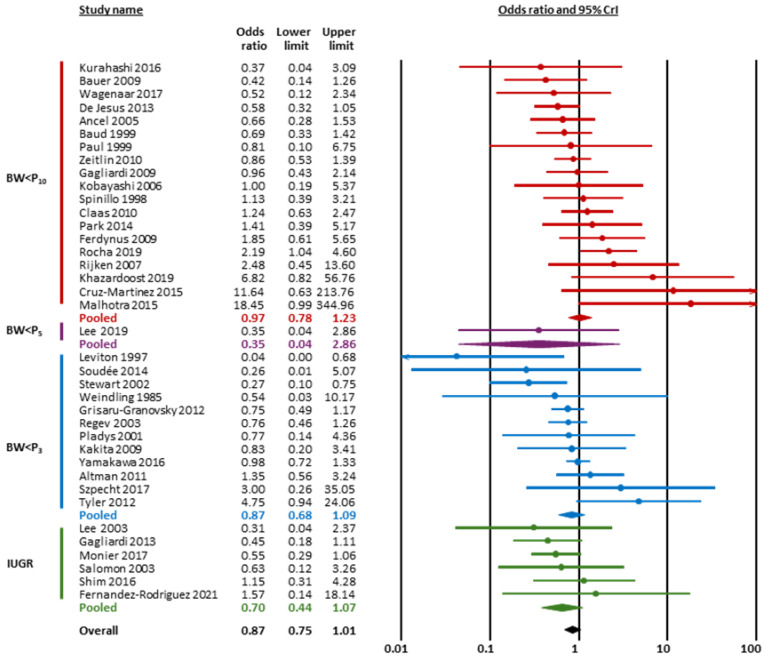
Bayesian model-averaged (BMA) meta-analysis of the association between fetal growth restriction and cystic periventricular leukomalacia. BW, birth weight; CrI, credible interval, IUGR, intrauterine growth restriction; P3, 3rd percentile; P10, 10th percentile; SGA, small for gestational age [40,41,42,43,47,48,49,52,53,54,56,57,59,60,61,64,66,68,69,71,72,77,79,81,83,84,86,89,91,92,93,94,96,97,99,102,103,104].

Figure 2 and Table 1 summarize the results of the BMA meta-analysis. LogOR was converted to OR for clarity. BMA analysis showed strong evidence in favor of a positive association between chorioamnionitis and cPVL (OR 1.58, 95% CrI 1.12 to 2.20, BF_10_ = 20.51, Figure 2 and Figure 3) and extreme evidence in favor of a negative association between HDPs and cPVL (OR 0.63, 95% CrI 0.54 to 0.75, BF_10_ = 2936.82, Figure 2 and Figure 4) (Table 1). The evidence in favor of heterogeneity was extreme for the meta-analysis of chorioamnionitis (BF_rf_ > 10^6^) and inconclusive for the meta-analysis of HDPs (BF_rf_ = 0.88). The RoBMA showed moderate evidence against publication bias in the HDP analysis (BF_bias_ = 0.21) and inconclusive evidence against publication bias in the chorioamnionitis analysis (BF_bias_ = 0.58) (Table S2). Regarding SGA/IUGR, the BMA analysis showed inconclusive evidence in favor of a negative association with cPVL (OR 0.87, 95% CrI 0.75 to 1.01, BF_10_ = 1.41, Figure 2 and Figure 5). The evidence for heterogeneity was inconclusive (BF_rf_ =0.68, Table 1) and the RoBMA showed inconclusive evidence against publication bias for this analysis (BF_bias_ = 0.53) (Table S2).

Regarding GA, the BMA analysis showed extreme evidence in favor of a lower GA of the chorioamnionitis-exposed infants when compared with the control group (BF_10_ = 226.52, Table 2). In contrast, the HDP group had a higher GA than the corresponding control group and the BMA analysis showed that the evidence in favor of this difference in GA was moderate. (BF_10_ = 9.78, Table 2). The BMA analysis showed inconclusive evidence in favor or against GA differences in the SGA/IUGR meta-analysis (BF_10_ = 0.93, Table 2).

### 3.3. Subgroup Analysis and Meta-Regression

Subgroup analyses based on the subtype of chorioamnionitis showed that the evidence in favor of a positive association with cPVL was moderate for any chorioamnionitis (BF_10_ = 4.44,) and clinical chorioamnionitis (BF_10_ = 3.93) but inconclusive for histological chorioamnionitis (BF_10_ = 1.15) (Figure 2, Table 1). Regarding funisitis, the BMA analysis showed inconclusive evidence against an association with cPVL regardless of the control group (infants without funisitis, infants without funisitis but with chorioamnionitis, and infants without funisitis or chorioamnionitis) (Table 1).

Subgroup analyses based on the subtype of HDP showed that the evidence in favor of a negative association with cPVL was extreme for any HDP (BF_10_ = 196.19), moderate for preeclampsia (BF_10_ = 3.25), and weak for preeclampsia/HELLP (BF_10_ = 2.82) (Table 1). Subgroup analyses based on the subtype of SGA/IUGR showed that the evidence was weak/inconclusive for the three subgroups (BW < P10, BW < P3, and IUGR) (Table 1).

Meta-regression showed that the differences in GA between the infants exposed or unexposed to chorioamnionitis, HDPs, or growth retardation in each individual study did not correlate with the effect size of the association between the condition and cPVL (1/3 < BF_mod_ < 3) (Supplementary Table S3).

## 4. Discussion

Neonatal outcomes after very preterm birth are contingent upon both immaturity, as measured by low GA, and the pathological conditions that result in early delivery. The present Bayesian meta-analysis showed an association between the inflammatory/infectious endotype, represented by chorioamnionitis, and increased odds of developing cPVL. The strength of the evidence on the association between chorioamnionitis and cPVL was higher for clinical than for histologic chorioamnionitis. Conversely, the placental dysfunction endotype, as represented by HDPs, was associated with decreased odds of developing cPVL. Furthermore, the Bayesian analysis showed that the evidence in favor or against an association between SGA/IUGR and cPVL was inconclusive.

In a previous frequentist meta-analysis, Abiramalatha et al. exhaustively investigated the association of PVL with different pre- and postnatal risk factors [6]. Their results are in agreement with ours for the three conditions that we have analyzed. The main novelties of our meta-analysis are the use of Bayesian statistics and that our analysis focused exclusively on cPVL, whereas Abiramalatha et al. combined non-cystic and cPVL in their meta-analysis [6]. The use of a Bayesian approach provides a more nuanced insight into the strength of evidence by quantifying the relative support for H_1_ (presence of effect/association) and H_0_ (absence of effect/association) given the data. When using frequentist H_0_ significance testing (NHST), a *p*-value greater than 0.05 or a confidence interval crossing the line of no effect indicate that the data do not provide strong evidence against H_0_, but it does not confirm that H_0_ should be accepted [105,106]. In contrast, the BF_10_ compares the likelihood of the data under H_1_ to the likelihood under H_0_. A BF_10_ close to 1 indicates that the data do not strongly favor either hypothesis, suggesting an absence of evidence [105,106]. Conversely, a BF_10_ markedly greater than 1 or less than 1 provides evidence in favor of one hypothesis over the other and may indicate evidence of no effect if H_0_ is strongly supported. This is particularly useful in distinguishing between absence of evidence and evidence of absence [105,106,107]. For example, in the SGA/IUGR meta-analysis, the frequentist conclusion would be that there is no “statistically significant” association with cPVL, but we cannot be certain that there is truly evidence of no association. In contrast, the BF_10_ of 1.41 allow us to ascertain the data were only 1.41 times more likely under the presence-of-association hypothesis in comparison to the absence of association hypothesis. In other words, the evidence provided by the SGA/IUGR data is inconclusive.

In contrast to the SGA/IUGR data, the respective BF_10_ for chorioamnionitis and HDPs were 20.5 and 2937. Thus, the chorioamnionitis and HDP data were, respectively, 20.5 and 2937 times more likely under the under the presence of association hypothesis in comparison to the absence of association hypothesis. These data provide conclusive evidence of an association between the two prenatal conditions and the risk of developing cPVL. According to the categories proposed by Lee and Wagenmakers [34], the evidence was categorized as strong in favor of H_1_ for chorioamnionitis (BF_10_ from >10 to 30), and as extreme in favor of H_1_ for HDPs (BF_10_ > 100). Notably, the association was diametrically opposed, with chorioamnionitis increasing the risk and HDPs decreasing the risk of cPVL. However, association does not imply causation and the effect of chorioamnionitis and HDPs on the risk of cPVL may be mediated more by their role as preterm birth triggers than by the different pathophysiologic conditions that accompany the two conditions. Moreover, prenatal insults, such as chorioamnionitis or placental insufficiency, may have an additive or synergistic effect when combined with postnatal insults, such as hypoxia, hyperoxia, oxidative stress, or postnatal infection/inflammation. It should also be noted that since very or extremely preterm birth is by definition a pathological condition, there is no “healthy control” group for comparison. Thus, the lower risk of developing cPVL in the HDP-exposed group may be the consequence of the lower rate of chorioamnionitis in this group. In addition, infants exposed to chorioamnionitis had lower GA and infants exposed to HDPs had higher GA than their respective controls. However, meta-regression did not provide conclusive evidence that these differences in GA were correlated with the effect size of the association between cPVL and chorioamnionitis, HDPs, or SGA/IUGR. This is in contrast to other adverse outcomes of prematurity, such as BPD, ROP, LOS, or mortality, for which meta-regression showed a correlation of the exposure–outcome association with differences in GA between the insult-exposed and non-exposed groups [19,20,21,23,24].

Beyond the disparities in GA, the two endotypes of prematurity may also exhibit variations in the frequency of prenatal interventions, such as the administration of magnesium sulfate for neuroprotection or antenatal corticosteroids, which have been demonstrated to moderate the risk of neonatal brain injury [108,109]. Finally, an important limitation of our meta-analysis is the dichotomous characterization of the different pathological conditions, which does not take into account the intensity and duration of the prenatal insults.

The inflammatory response associated with pre- and postnatal infections can lead to the activation of microglia and the release of pro-inflammatory cytokines such as tumor necrosis factor (TNF)-alpha and interleukin (IL)-6. These cytokines can cause direct damage to oligodendrocyte precursors, which are crucial for myelination, and also can disrupt cerebral hemodynamics resulting in hypoxic–ischemic injury to the white matter [1,9,10,11]. The association between chorioamnionitis and PVL has been demonstrated in numerous cohorts and meta-analyses [6,110]. Interestingly, we observed that the effect size and strength of evidence for the association between chorioamnionitis and cPVL is greater for clinical than for histological chorioamnionitis. Clinical chorioamnionitis is a clinical diagnosis made during labor, based on maternal fever plus additional signs such as maternal or fetal tachycardia, uterine tenderness, purulent or malodorous vaginal discharge, or maternal leukocytosis [111,112]. Histological chorioamnionitis is a pathological diagnosis made by microscopic examination of the placenta, membranes, and umbilical cord, characterized by neutrophilic infiltration of the chorion, amnion, or both. It reflects a maternal inflammatory response to chemotactic signals in the amniotic cavity and may be present with or without clinical symptoms [111,112]. Therefore, clinical chorioamnionitis may represent a more acute and severe inflammatory state, which may have a more profound impact on fetal brain development. In contrast, histological chorioamnionitis may represent a more chronic or subclinical inflammatory state, which still contribute to white matter injury but with less immediate severity [10,109,112]. Nevertheless, it should be noted that clinical chorioamnionitis encompasses a broad spectrum of conditions characterized by infection, inflammation, or both and that the majority of clinical signs linked with clinical chorioamnionitis are not specific. This heterogeneity results in inconsistent clinical presentations and diagnostic criteria [111,112].

The differences between clinical and histological chorioamnionitis also entail differences in perinatal clinical management. Preterm infants exposed to clinical chorioamnionitis require immediate sepsis evaluation and empiric antibiotics, while those with isolated histological chorioamnionitis require close monitoring but not routine antibiotics unless clinically indicated [113]. In addition, incorporating routine placental analysis, including histological examination for chorioamnionitis and fetal inflammatory response, into the management of preterm infants is increasingly recognized as clinically valuable, particularly for very preterm births However, practical implementation requires timely placental examination and effective communication between pathology and neonatal teams [114].

An additional noteworthy finding of our meta-analysis was that the evidence on the association between funisitis and cPVL was also inconclusive. Funisitis is closely associated with the fetal inflammatory response syndrome, which is characterized by elevated levels of inflammatory cytokines [115,116]. In a previous Bayesian meta-analysis, we analyzed the potential association of funisitis with short-term outcomes of prematurity including PVL, IVH, BPD, ROP, LOS, and mortality [28]. We observed that the evidence in favor of an association between funisitis and risk of developing the outcome was conclusive (BF_10_ > 3) only for IVH and LOS. Moreover, when the control group was restricted to infants having chorioamnionitis without funisitis, the only outcome associated with funisitis was IVH. These findings suggest that the presence of funisitis does not appear to augment the risk of most adverse preterm birth outcomes when compared to chorioamnionitis in the absence of a fetal inflammatory response [28].

With regard to HDPs, the Bayesian analysis showed extreme evidence of association with a reduced risk of developing cPVL (BF_10_ = 2937). Accordingly, some researchers have suggested that HDPs may play a protective role in the complications associated with prematurity, including severe brain damage [42,117]. However, the independent risk attributable to HDPs on premature brain development is controversial, with studies showing increased, decreased, or unchanged risk of PVL in infants exposed to HDPs [117,118,119,120]. One possible reason for the inconsistent findings is the variation in the severity of HDPs. It has been suggested that milder phenotypes of HDPs may be protective through a preconditioning effect on the fetal brain, resulting in reduced brain susceptibility to subsequent more severe hypoxic–ischemic insults [117]. In addition, some studies demonstrated no association between HDP exposure and neurodevelopment in infants born preterm, when controlling for differences in GA, BW, or other potential confounders [119,120]. Furthermore, the meta-analysis by Abiramalatha et al. does not report an association between HDPs and PVL, because this was only observed when considering the unadjusted odds ratio, not when adjusting for potential confounders [6]. This is another relevant difference between our meta-analysis and that of Abiramalatha et al. [6]. They prioritized the use of adjusted odds ratios, whereas we used unadjusted odds ratios.

In meta-analyses, it is a common practice to prioritize adjusted effect sizes over unadjusted ones because adjustment is generally intended to control for confounding factors and may provide a more accurate estimate of the exposure–outcome association. This approach is embedded in quality assessment tools, such as the Newcastle–Ottawa Scale, which awards higher scores to studies that report adjusted estimates [29]. The rationale is that confounding is usually a major threat to validity in epidemiological contexts, and statistical adjustment is presumed to mitigate this risk. However, in perinatal epidemiology, this practice raises significant methodological concerns when the adjustment set includes variables such as the GA, which is a mediator on the causal pathway between prenatal exposures and outcomes of prematurity, such as cPVL. Adjusting for GA in this context can introduce overadjustment and collider bias, distorting or even reversing the true association between exposure and outcome [121,122]. This is because GA is influenced by the exposure and itself influences the outcome, so conditioning on it can block part of the causal effect and induce spurious associations [121,122]. In addition, in meta-analyses of observational studies, heterogeneity in the set of confounders used across studies and the frequent lack of justification for confounder selection are major sources of bias and imprecision. In a substantial proportion of the observational studies included in meta-analyses the selection of confounders is not justified or relies on statistical criteria, rather than explicit causal reasoning [123]. This practice is problematic because statistical significance alone does not distinguish true confounders from mediators or colliders and can lead to inappropriate adjustment. Confounder selection should be grounded in causal inference principles, ideally using directed acyclic graphs or similar frameworks, to ensure valid effect estimation and comparability across studies [123]. The combination of all the aforementioned factors leads to the conclusion that, at least in meta-analyses of perinatal epidemiology, the use of crude or least adjusted estimates is necessary to maximize comparability and to avoid bias from inappropriate adjustment for mediators. Stratified or sensitivity analyses can be performed to explore the impact of adjustment, but the main analysis should not combine effect estimates that are not conceptually equivalent.

HDPs are closely linked with maternal vascular malperfusion, which in turn contributes to the development of fetal growth retardation [124]. The gradation of HDPs, from mild gestational hypertension to severe preeclampsia, correlates with the extent of chronic fetal oxygen deficiency and its impact on fetal growth and development [124,125]. The Bayesian analysis showed that the evidence for an association with cPVL was inconclusive for SGA/IUGR, whereas it was extreme for HDPs. This may suggest that the reduced risk of developing cPVL associated with HDPs is not present when the insult is severe enough to induce growth retardation because the fetus is then exposed to more severe chronic hypoxia. Similarly, in a previous meta-analysis, we observed that HDPs were associated with decreased risk of mortality, whereas SGA/IUGR was associated with an increased risk of mortality in very preterm infants [16]. However, our meta-analysis is limited because most of the included studies are based on SGA infants. SGA differs from IUGR principally because it also encompasses a number of constitutionally small but healthy fetuses at lower risk of adverse outcomes. On the other hand, growth-restricted fetuses with BW > 10th centile may not meet their growth potential and may remain undiagnosed despite being at increased risk of adverse outcome [27]. Failure to differentiate these entities can confound research outcomes, clinical management, and prognostication. For example, studies grouping SGA and IUGR together may obscure the specific risks and mechanisms associated with true growth restriction, as SGA infants without IUGR do not share the same risk profile for adverse outcomes [27]. Including the distinction between IUGR and SGA in future research will improve the accuracy of epidemiological studies, clarify the impact of antenatal insults versus constitutional factors, and facilitate the development of personalized care plans for affected infants.

Our meta-analysis focused on cPVL because it is the form of white matter injury associated with worse outcome and because most studies used ultrasonography for the assessment of withe matter injury. While major intracranial lesions can be reliably diagnosed with sequential cranial ultrasonography, more subtle lesions, especially those involving the white matter, may be overlooked or may not be detectable with this imaging technique and the diagnosis of subtle white matter lesions remains subjective [3,5,126,127,128,129]. However, there is an increasing recognition that cPVL is no longer a prevalent finding, and non-cystic PVL has emerged as the predominant form of white matter abnormality in very preterm infants [3,5,126,127,128,129]. It has been suggested that improvements in perinatal and neonatal care, including use of antenatal steroids, better ventilation strategies, and optimized nutritional support, have contributed to this decline in cPVL prevalence [3,5,129]. In addition, the increased use of MRI for neuroimaging has led to the detection of more subtle forms of white matter injury, shifting the focus from cPVL to these subtler lesions [3,5,129]. Unfortunately, a consensus classification system for PVL based on MRI does not currently exist, which hinders the dichotomization required for meta-analysis. As the utilization of MRI expands and the capacity to discern minimal white matter injuries improves, clinical practice and research will be compelled to adapt by incorporating MRI findings into risk stratification, prognostication, and outcome prediction models. This shift necessitates the development of novel classification systems for white matter injury, the establishment of longitudinal imaging protocols, and the meticulous interpretation of the clinical significance of subtle MRI abnormalities, as a significant proportion of these abnormalities do not correlate with severe neurodevelopmental outcomes [5,129].

In conclusion, the present Bayesian meta-analysis provides evidence indicative of an association between antenatal infection–inflammation and an increased risk of developing cPVL in preterm infants. Conversely, the likelihood of developing cPVL is diminished in infants exposed to HDPs. Further research is warranted to ascertain whether the associations identified for cPVL are also present for more subtle forms of white matter injury. The present results underscore the importance of considering the pathologic condition, or endotype, that triggers preterm birth in the prognosis of prematurity.

## Figures and Tables

**Table 1:** Bayesian model-averaged (BMA) meta-analysis of the association between endotype of prematurity and cystic periventricular leukomalacia.

Condition	Subgroup	k	OR	95% CrI		Tau	95% CrI		BF_10_	BF_rf_	Evidence for Effect	Evidence for Heterogeneity
				Lower Limit	Upper Limit		Lower Limit	Upper Limit				
Chorioamnionitis	Any	3	1.83	0.87	3.87	1.75	1.10	5.77	4.44	1.69	moderate for	undecided for
	Clinical	14	1.67	0.96	3.00	2.97	2.00	5.46	3.93	>10^6^	moderate for	extreme for
	Histological	17	1.18	0.96	1.47	1.30	1.09	1.75	1.15	1.89	undecided for	undecided for
	Overall	34	1.58	1.12	2.20	2.25	1.79	3.05	20.51	>10^6^	strong for	extreme for
Funisitis	Fun+ vs. Fun−/CA−	6	0.97	0.68	1.39	1.26	1.07	1.84	0.55	0.56	undecided against	undecided against
	Fun+ vs. Fun−/CA+	4	0.88	0.55	1.35	1.34	1.08	2.44	0.76	0.75	undecided against	undecided against
	Fun+ vs. Fun−	5	0.93	0.65	1.33	1.26	1.08	1.85	0.60	0.53	undecided against	undecided against
Hypertensive disorders of pregnancy	Any	14	0.66	0.54	0.80	1.20	1.07	1.55	196.19	0.32	extreme for	moderate against
	Preeclampsia	15	0.75	0.56	1.00	1.32	1.08	1.92	3.25	0.92	moderate for	undecided against
	Preeclampsia/HELLP	2	0.65	0.40	1.22	1.65	1.12	4.19	2.82	3.89	undecided for	moderate for
	Overall	31	0.63	0.54	0.75	1.21	1.08	1.47	2936.82	0.88	extreme for	undecided against
SGA/IUGR	BW < P10	19	0.97	0.78	1.23	1.27	1.08	1.69	0.37	0.82	undecided against	undecided against
	BW < P3	11	0.87	0.68	1.09	1.28	1.08	1.91	0.84	0.63	undecided against	undecided against
	IUGR	6	0.70	0.44	1.07	1.34	1.08	2.28	2.41	0.85	undecided for	undecided against
	Overall	37	0.87	0.75	1.01	1.24	1.08	1.56	1.41	0.68	undecided for	undecided against

BF: Bayes factor; CrI: credible interval; Fun+: presence of funisitis; Fun−: absence of funisitis regardless of the state of chorioamnionitis; Fun−/CA−: absence of funisitis and chorioamnionitis; Fun−/CA+: absence of funisitis but presence of chorioamnionitis; IUGR: intrauterine growth restriction; K: number of studies; SGA: small for gestational age.

**Table 2:** Bayesian model-averaged (BMA) meta-analysis of the differences in GA (mean GA of exposed group minus mean GA of non-exposed group of each individual study) in chorioamnionitis, hypertensive disorders of pregnancy, and SGA/IUGR.

Condition	k	GA Difference (Hedges’ *g*)	95% CrI		Tau	95% CrI		BF_10_	BF_rf_	Evidence for Effect	Evidence for Heterogeneity
			Lower Limit	Upper Limit		Lower Limit	Upper Limit				
Chorioamnionitis	14	−0.52	−0.75	−0.27	0.44	0.30	0.66	226.52	>10^6^	extreme for	extreme for
Hypertensive disorders of pregnancy	7	0.37	0.10	0.62	0.35	0.19	0.67	9.78	>10^6^	moderate for	extreme for
SGA/IUGR	12	0.21	−0.12	0.54	0.62	0.39	0.97	0.93	>10^6^	undecided against	extreme for

BF: Bayes factor; CrI: credible interval; GA: gestational age; IUGR: intrauterine growth restriction; K: number of studies; SGA: small for gestational age.

## Data Availability

All data relevant to the study are included in the article or uploaded as Supplementary Information. Additional data are available upon reasonable request.

## Supplementary Materials

The following supporting information can be downloaded at: https://www.mdpi.com/article/10.3390/children12081065/s1, File S1: Search strategy; Table S1: Characteristics of the included studies and risk of bias assessment; Table S2: Robust Bayesian meta-analysis (RoBMA) on the association between endotype of prematurity and cystic periventricular leukomalacia; Table S3: Bayesian model-averaged meta-regression (BMA-reg) of the moderating effect of the difference in GA (mean GA of exposed group minus mean GA of non-exposed group of each individual study) on the association between endotype of prematurity and cystic periventricular leukomalacia. Refs. [130,131,132,133] are listed in Supplementary Materials.
