# Supplementary material for: Association Between Endotype of Prematurity and Cystic Periventricular Leukomalacia: A Bayesian Model-Averaged Meta-Analysis
Source: Children (Basel). 2025 Aug 13;12(8):1065. doi: 10.3390/children12081065 (PMC12384824; doi:10.3390/children12081065)
Supplement: Supplementary file 1 [file children-12-01065-s001.zip › children-3760718-supplementary.pdf]

Supplementary Online Content

Association between Endotype of Prematurity and Cystic Periventricular Leukomalacia: A Bayesian Model-Averaged Meta-Analysis

Neirude PA Lissone, Tamara M Hundscheid, Gloria Galán-Henríquez, František Bartoš, Eduardo Villamor

1. **Methods**

   1.1 Search strategy.

   1.2 Supplementary information on methods.

2. **Results**

   Table S1. Characteristics of the included studies and risk of bias assessment.

   Table S2. Robust Bayesian meta-analysis (RoBMA) on the association between endotype of prematurity and cystic periventricular leukomalacia

   Table S3. Bayesian model-averaged meta-regression (BMA-reg) of the moderating effect of the difference in GA (mean GA of exposed group minus mean GA of non-exposed group of each individual study) on the association between endotype of prematurity and cystic periventricular leukomalacia.

1. Methods

1.1. Search strategy

PubMed

(("Periventricular Leukomalacia"[MeSH Terms] OR "periventricular leukomalacia"[Title/Abstract] OR "periventricular leucomalacia"[Title/Abstract] OR "PVL"[Title/Abstract] OR ("White Matter"[MeSH Terms] AND ("injury"[Title/Abstract] OR "damage"[Title/Abstract] OR "lesion"[Title/Abstract])) OR "white matter injury"[Title/Abstract] OR "white matter damage"[Title/Abstract] OR "white matter lesion"[Title/Abstract]) AND (("Chorioamnionitis"[MeSH Terms] OR "chorioamnionitis"[Title/Abstract] OR "amnionitis"[Title/Abstract] OR "funisitis"[Title/Abstract] OR "maternal infection"[Title/Abstract] OR "intrauterine infection"[Title/Abstract]) OR ("Hypertension, Pregnancy-Induced"[MeSH Terms] OR "gestational hypertension"[Title/Abstract] OR "pregnancy-induced hypertension"[Title/Abstract] OR "gestational hypertensive disorders"[Title/Abstract] OR "Preeclampsia"[MeSH Terms] OR "preeclampsia"[Title/Abstract] OR "pre-eclampsia"[Title/Abstract] OR "HELLP Syndrome"[MeSH Terms] OR "HELLP syndrome"[Title/Abstract]) OR ("Fetal Growth Retardation"[MeSH Terms] OR "fetal growth restriction"[Title/Abstract] OR "FGR"[Title/Abstract] OR "intrauterine growth restriction"[Title/Abstract] OR "IUGR"[Title/Abstract] OR "Small for Gestational Age"[MeSH Terms] OR "small for gestational age"[Title/Abstract] OR "SGA"[Title/Abstract])) AND ("Infant, Premature"[MeSH Terms] OR "Infant, Newborn"[MeSH Terms] OR "neonate"[Title/Abstract] OR "neonatal"[Title/Abstract] OR "newborn"[Title/Abstract] OR "preterm"[Title/Abstract] OR "premature infant"[Title/Abstract]))

Embase

(('periventricular leukomalacia'/exp OR 'periventricular leukomalacia':ti,ab,kw OR 'periventricular leucomalacia':ti,ab,kw OR 'PVL':ti,ab,kw OR ('white matter'/exp AND ('injury':ti,ab,kw OR 'damage':ti,ab,kw OR 'lesion':ti,ab,kw)) OR 'white matter injury':ti,ab,kw OR 'white matter damage':ti,ab,kw OR 'white matter lesion':ti,ab,kw) AND (('chorioamnionitis'/exp OR 'chorioamnionitis':ti,ab,kw OR 'amnionitis':ti,ab,kw OR 'funisitis':ti,ab,kw OR 'maternal infection':ti,ab,kw OR 'intrauterine infection':ti,ab,kw) OR ('hypertension in pregnancy'/exp OR 'gestational hypertension':ti,ab,kw OR 'pregnancy-induced hypertension':ti,ab,kw OR 'gestational hypertensive disorders':ti,ab,kw OR 'preeclampsia'/exp OR 'preeclampsia':ti,ab,kw OR 'pre-eclampsia':ti,ab,kw OR 'HELLP syndrome'/exp OR 'HELLP syndrome':ti,ab,kw) OR ('fetal growth restriction'/exp OR 'fetal growth restriction':ti,ab,kw OR 'FGR':ti,ab,kw OR 'intrauterine growth restriction':ti,ab,kw OR 'IUGR':ti,ab,kw OR 'small for gestational age'/exp OR 'small for gestational age':ti,ab,kw OR 'SGA':ti,ab,kw)) AND ('premature infant'/exp OR 'newborn'/exp OR 'neonate':ti,ab,kw OR 'neonatal':ti,ab,kw OR 'newborn':ti,ab,kw OR 'preterm':ti,ab,kw OR 'premature infant':ti,ab,kw))

No language limits were set. Narrative reviews, systematic reviews, case reports, letters, editorials, and commentaries were excluded, but read to identify potential additional studies. Additional strategies to identify studies included manual review of reference lists from key articles that fulfilled our eligibility criteria, use of "related articles" feature in PubMed, and use of the "cited by" tool in Web of Science and Google scholar. Two reviewers independently screened the results of the searches, and included studies according to the inclusion criteria using EndNote (RRID:SCR_014001), using the methodology described by Bramer et al. (1)

1.2. Supplementary information on methods

Study selection

Studies were included if they examined preterm (GA<37 weeks) and/or very low birth weight (≤1500g) infants and reported data that could be used to measure the association between exposure to chorioamnionitis, hypertensive disorders of pregnancy (HDP) or small for gestational age (SGA)/intrauterine growth restriction (IUGR) and cystic periventricular leukomalacia (cPVL). Therefore, we selected observational studies in which

the exposure (chorioamnionitis, HDP or SGA/IUGR) was the independent variable and the outcome (cPVL) the dependent variable and vice versa. Studies that exclusively included late preterm infants (GA>34 weeks) or that combined preterm and term infants were excluded. Due to the high number of included studies, no additional efforts were made to clarify definitions or other data with the authors. Abstracts and unpublished studies were also excluded. To identify relevant studies, two reviewers (TH, NL) independently screened the results of the searches and applied inclusion criteria using a structured form. Discrepancies were resolved through discussion or consultation with a third reviewer (EV).

Data extraction and quality assessment

Data extracted from each study included citation information, language of publication, country of conducted research, time period of the study, study objectives, study design, inclusion/exclusion criteria, definition criteria for chorioamnionitis, HDP, IUGR, SGA and PVL, patient characteristics, and results (including raw numbers or summary statistics when raw numbers were not available). A study was categorized as a case-control when each case was matched with a control in a predetermined ratio (1:1, 1:2, 1:3, etc.). A study was considered a cohort study when it included, prospectively or retrospectively, a group of infants during a given period, regardless of the number of infants included. Therefore, small cohorts were also included.

Any definition of chorioamnionitis, HDP, IUGR, or SGA was accepted but we performed sub-group analysis based on the different definitions. When a study used more than one definition criteria for growth restriction, definitions based on assessment of fetal growth (i.e., IUGR) prevailed over definitions based on BW. When a study used different BW threshold percentiles to define SGA, data from the lowest percentile were included. When a study did not specify the threshold percentile used, it was grouped together with the studies that used the 10th percentile.

Methodological quality was assessed using the Newcastle-Ottawa Scale for cohort or case-control studies.(2) This scale uses a star rating system (range: 0–9 stars) scoring three aspects of the study: selection (0–4), comparability (0–2) and exposure/outcome (0–3). Two reviewers (TH, EV-M) independently assessed the methodological quality of each study. Discrepancies were resolved through discussion.

Robust Bayesian meta-analysis (RoBMA)

We used RoBMA to assess the robustness of the results to the potential presence of publication bias (3). RoBMA extends the Bayesian model-averaged meta-analysis by the two major publication bias adjustment techniques: selection models (adjusting for the publication bias operating on *p*-values) and precision-effect test and precision-effect estimate with standard errors (PET-PEESE, adjusting for the relationship between effect sizes and standard errors) (4). The resulting RoBMA ensemble contains 36 models composed of the following assumptions about the presence vs. absence of the effect (2) x presence vs. absence of between-study heterogeneity (2) x presence vs. absence of publication bias adjustment models (6 selection models, PET, PEESE, and no bias). We used RoBMA with the same prior distributions for the effect and heterogeneity as in BMA and the default prior distributions for the publication bias adjustment part. Publication bias was expressed as BF_{bias} using the same categories for evidence previously described for BF_{10} and BF_{rf} (5).

2. Results

Table S1. Characteristics of the included studies and risk of bias assessment.

First author and publication year	Country	Study design	Prospective?	Mean GA of cohort (weeks)	Independent variable	Infants (n)	Study centers (n)	Selection	Comparability	Exposure/Outcome	NOS Total
Ahn 2012 (6)	South Korea	cohort	yes	30.6	Exposure	257	1	4	1	3	8
Alexander 1998 (7)	USA	cohort	yes	28.9	Exposure	1367	1	4	2	3	9
Altman 2011 (8)	Sweden	cohort	yes	33.1	Exposure	6674	NA	4	0	3	7
Ancel 2005 (9)	France	cohort	no	NA	PVL	1807	447	4	2	3	9
Baud 2000 (10)	France	cohort	yes	29.8	Exposure	685	1	4	1	3	8
Bauer 2009 (11)	Austria	cohort	no	NA	PVL	340	1	4	0	3	7
Been 2009 (12)	Netherlands	cohort	yes	30.7	Exposure	301	1	4	2	3	9
Bossung 2020 (13)	Germany	cohort	no	28.2	Exposure	16035	62	4	2	3	9
Botet 2011 (14)	Spain	ca-co	yes	28.3	Exposure	328	1	4	1	3	8
Claas 2010 (15)	Netherlands	cohort	no	30.0	Exposure	146	1	4	0	3	7
Cruz-Martinez 2015 (16)	Spain	cohort	yes	31.1	Exposure	180	1	4	0	3	7
De Jesus 2013 (17)	USA	cohort	no	25.0	Exposure	2971	17	4	2	3	9
Dempsey 2005 (18)	Canada	cohort	yes	27.0	Exposure	330	1	4	1	3	8
Denzler 2014 (19)	Switzerland	cohort	no	NA	PVL	6472	1	4	0	3	7

Ferdynus 2009 (20)	France	cohort	yes	30.5	Exposure	572	18	4	2	3	9
Fernandez-Rodriguez 2021 (21)	Spain	cohort	no	31.4	Exposure	94	1	4	2	3	9
Gagliardi 2009 (22)	Italy	cohort	yes	NA	PVL	1209	14	4	2	3	7
Gagliardi 2013 (23)	Italy	cohort	yes	28.4	Exposure	2085	51	4	2	3	9
Gagliardi 2014 (24)	Italy	cohort	no	27.3	Exposure	3606	82	4	2	3	9
Grisaru-Granovsky 2012 (25)	Israel	cohort	yes	28.2	Exposure	9756	28	4	2	3	9
Hendson 2011 (26)	Canada	cohort	yes	26.9	Exposure	628	1	4	1	3	8
Kakita 2009 (27)	Japan	ca-co	yes	NA	PVL	44	1	4	0	3	7
Khazardoost 2019 (28)	Iran	cohort	yes	31.8	Exposure	158	3	4	1	3	8
Kobayashi 2006 (29)	Japan	cohort	no	NA	PVL	64	7	4	0	3	7
Kubota 2001 (30)	Japan	cohort	no	NA	PVL	51	1	4	0	3	7
Kumazaki 2002 (31)	Japan	cohort	no	NA	PVL	127	1	4	0	3	7
Kurahashi 2016 (32)	Japan	cohort	no	NA	PVL	124	2	4	0	3	7
Lee 2002 (33)	South Korea	cohort	no	NA	PVL	88	1	4	0	3	7
Lee 2003 (34)	South Korea	cohort	no	NA	PVL	321	1	4	0	3	7
Lee 2019 Perinatology (35)	South Korea	cohort	no	28.1	PVL	126	1	4	2	3	9
Lee 2019 PLOS ONE (36)	South Korea	cohort	no	28.4	Exposure	2900	60	4	2	3	9
Leviton 1997 (37)	USA	cohort	yes	NA	PVL	1831	5	4	0	3	7
Maisonneuve 2017 (38)	France	cohort	yes	27.1	Exposure	3040	447	4	2	3	9
Malhotra 2015 (39)	Australia	cohort	yes	28.2	Exposure	459	1	4	2	3	9
Monier 2017 (40)	France	cohort	no	28.2	Exposure	2919	447	4	2	3	9
Morsing 2018 (41)	Sweden	cohort	no	26.1	Exposure	1152	1	4	0	3	7

Murata 2005 (42)	Japan	cohort	no	NA	PVL	201	1	4	0	3	7
Oda 2008 (43)	Japan	cohort	no	NA	PVL	30	1	4	0	3	7
Pappas 2014 (44)	USA	cohort	no	25.7	Exposure	2390	16	4	1	3	8
Park 2014 (45)	South Korea	cohort	no	NA	PVL	84	1	4	1	3	8
Paul 1998 (46)	USA	cohort	no	28.7	Exposure	365	1	4	0	3	7
Paul 1999 (47)	USA	cohort	no	NA	PVL	176	1	4	0	3	7
Perlman 1996 (48)	USA	cohort	no	NA	PVL	632	1	4	0	3	7
Pladys 2001 (49)	France	cohort	yes	NA	PVL	46	1	4	0	3	7
Regev 2003 (50)	Israel	cohort	no	28.0	Exposure	2764	28	4	0	3	7
Regev 2015 (51)	Israel	cohort	yes	29.9	Exposure	2139	28	4	2	3	9
Rijken 2007 (52)	Netherlands	cohort	yes	17.2	Exposure	266	3	4	0	3	7
Rocha 2006 (53)	Portugal	cohort	no	29.4	Exposure	452	3	4	1	3	8
Rocha 2018 (54)	Portugal	cohort	yes	27.2	Exposure	494	11	4	1	3	8
Rocha 2019 (55)	Portugal	cohort	no	27.6	Exposure	494	11	4	1	3	8
Ryan 2020 (56)	Ireland	cohort	no	28.5	Exposure	499	1	4	1	3	8
Salomon 2003 (57)	France	cohort	yes	NA	PVL	66	2	4	2	3	9
Shankaran 2006 (58)	USA	cohort	yes	NA	PVL	778	16	4	2	3	9
Shim 2016 (59)	South Korea	cohort	no	NA	PVL	172	1	4	1	3	8
Soudée 2014 (60)	France	cohort	yes	30.0	Exposure	150	1	4	2	3	9
Spinillo 1998 (61)	Italy	cohort	no	NA	PVL	349	1	4	0	3	7
Stewart 2002 (62)	Usa	cohort	no	NA	PVL	1419	5	4	0	3	7
Tokumasu 2016 (63)	Japan	cohort	no	25.9	Exposure	4518	131	4	0	3	7

Tyler 2012 (64)	USA	cohort	no	NA	PVL	877	14	4	0	3	7
Wagenaar 2017 (65)	International	cohort	no	NA	PVL	250	2	4	1	3	8
Wang 2017 (66)	Taiwan	cohort	no	NA	PVL	4919	22	4	1	3	8
Weindling 1985 (67)	UK	cohort	yes	NA	PVL	86	1	4	0	3	7
Wiswell 1996 (68)	USA	cohort	yes	NA	PVL	58	1	4	0	3	7
Wu 2023 (69)	Taiwan	cohort	yes	29.0	PVL	976	4	4	2	3	9
Yamakawa 2016 (70)	Japan	cohort	no	25.6	Exposure	9149	89	4	2	3	9
Zeitlin 2010 (71)	International	cohort	no	28.5	Exposure	4525	NA	4	2	3	9

Ca-co: case-control study; NA: not available GA: gestational age; NOS: Newcastle-Ottawa scale; USA: United States of America.

Table S2. Robust Bayesian meta-analysis (RoBMA) on the association between endotype of prematurity and cystic periventricular leukomalacia

Condition	Subgroup	k	OR	95% CrI		Tau	95% CrI		BF_{10}	BF_{rf}	BF_{bias}
				Lower limit	Upper limit		Lower limit	Upper limit			
Chorioamnionitis	Any	3	1.61	0.81	3.57	1.65	1.09	4.68	2.36	1.63	1.75
	Clinical	14	1.22	0.58	2.61	3.18	2.12	5.73	1.24	$>10^6$	3.65
	Histological	17	1.14	0.89	1.45	1.28	1.09	1.72	0.72	1.26	1.03
	Overall	34	1.49	0.93	2.17	2.30	1.81	3.18	4.36	$>10^6$	0.58
Funisitis	Fun+ vs Fun-/CA-	6	0,99	0,69	1,43	1,26	1,07	1,84	0,56	0,55	0,31
	Fun+ vs Fun-/CA+	4	0,91	0,56	1,43	1,34	1,08	2,39	0,73	0,73	0,63
	Fun+ vs Fun-	5	1,03	0,68	1,68	1,26	1,07	1,84	0,67	0,52	1,14
Hypertensive disorders of pregnancy	Any	14	0.67	0.55	0.85	1.21	1.07	1.57	32.99	0.34	0.35
	Preeclampsia	15	0.80	0.58	1.17	1.30	1.08	1.88	1.36	0.81	1.51
	Preeclampsia/HELLP	2	0.69	0.41	1.32	1.65	1.12	4.05	2.10	4.98	0.91
	Overall	31	0.64	0.54	0.79	1.22	1.08	1.51	113.00	0.96	0.21
SGA/IUGR	BW<P10	19	0.98	0.79	1.24	1.26	1.08	1.68	0.37	0.78	0.24
	BW<P3	11	0.91	0.70	1.21	1.26	1.08	1.86	0.59	0.54	1.44
	IUGR	6	0.74	0.45	1.17	1.33	1.08	2.18	1.73	0.83	0.73
	Overall	37	0.88	0.75	1.04	1.23	1.07	1.55	1.02	0.62	0.53

BF: Bayes factor; BW: birth weight; CrI: credible interval; Fun+: presence of funisitis; Fun−: absence of funisitis regardless of the state of chorioamnionitis; Fun−/CA−: absence of funisitis and chorioamnionitis; Fun−/CA+: absence of funisitis but presence of chorioamnionitis; IUGR: intrauterine growth restriction; K: number of studies; SGA: small for gestational age.

Table S3. Bayesian model-averaged meta-regression (BMA-reg) of the moderating effect of the difference in GA (mean GA of exposed group minus mean GA of non-exposed group of each individual study) on the association between endotype of prematurity and cystic periventricular leukomalacia.

Prior distribution scaled to	Condition	k	BF_{10}	BF_{rf}	BF_{mod}	Evidence for effect	Evidence for heterogeneity	Evidence for moderation
1/2 of the mean effect size	Chorioamnionitis	14	5.15	73.91	1.00	moderate for	very strong for	undecided for
	Hypertensive disorders of pregnancy	7	46.97	1.04	0.59	very strong for	undecided for	undecided against
	SGA/IUGR	12	0.43	1.14	0.70	undecided against	undecided for	undecided against
1/4 of the mean effect size	Chorioamnionitis	14	5.24	68.53	1.04	moderate for	very strong for	undecided for
	Hypertensive disorders of pregnancy	7	50.75	1.00	0.81	very strong for	undecided against	undecided against
	SGA/IUGR	12	0.42	1.11	0.89	undecided against	undecided for	undecided against

BF: Bayes factor; GA: gestational age; IUGR: intrauterine growth restriction; SGA: small for gestational age.

References

1. Bramer WM, Milic J, Mast F. Reviewing retrieved references for inclusion in systematic reviews using EndNote. J Med Libr Assoc. 2017;105(1):84-7.
2. Wells GA, Shea B, O'Connell D, Peterson J, Welch V, Losos M, et al. The Newcastle-Ottawa Scale (NOS) for assessing the quality of nonrandomised studies in meta-analyses. 2000.
3. Bartoš F, Maier M, Wagenmakers EJ, Doucouliagos H, Stanley T. Robust Bayesian meta-analysis: Model-averaging across complementary publication bias adjustment methods. Research Synthesis Methods. 2023;14(1):99-116.
4. Stanley TD, Doucouliagos H. Meta-regression approximations to reduce publication selection bias. Research Synthesis Methods. 2014;5(1):60-78.
5. Lee M, Wagenmakers E-J. Bayesian data analysis for cognitive science: A practical course. New York, NY: Cambridge University Press; 2013.
6. Ahn HM, Park EA, Cho SJ, Kim Y-J, Park H-S. The association of histological chorioamnionitis and antenatal steroids on neonatal outcome in preterm infants born at less than thirty-four weeks' gestation. Neonatology. 2012;102(4):259-64.
7. Alexander JM, Gilstrap LC, Cox SM, Leveno KJ. Clinical chorioamnionitis and the prognosis for very low birth weight infants. Obstetrics & Gynecology. 1998;91(5):725-9.
8. Altman M, Vanpée M, Cnattingius S, Norman M. Neonatal morbidity in moderately preterm infants: a Swedish national population-based study. The Journal of pediatrics. 2011;158(2):239-44. e1.
9. Ancel P-Y, Marret S, Larroque B, Arnaud C, Zupan-Simunek V, Voyer M, et al. Are maternal hypertension and small-for-gestational age risk factors for severe intraventricular hemorrhage and cystic periventricular leukomalacia? Results of the EPIPAGE cohort study. American journal of obstetrics and gynecology. 2005;193(1):178-84.
10. Baud O, Zupan V, Lacaze-Masmonteil T, Audibert F, Shojaei T, Thebaud B, et al. The relationships between antenatal management, the cause of delivery and neonatal outcome in a large cohort of very preterm singleton infants. BJOG: An International Journal of Obstetrics & Gynaecology. 2000;107(7):877-84.
11. Bauer M, Fast C, Haas J, Resch B, Lang U, Pertl B. Cystic periventricular leukomalacia in preterm infants: an analysis of obstetric risk factors. Early human development. 2009;85(3):163-9.
12. Been JV, Rours IG, Kornelisse RF, Passos VL, Kramer BW, Schneider TA, et al. Histologic chorioamnionitis, fetal involvement, and antenatal steroids: effects on neonatal outcome in preterm infants. American journal of obstetrics and gynecology. 2009;201(6):587. e1-. e8.
13. Bossung V, Fortmann MI, Fusch C, Rausch T, Herting E, Swoboda I, et al. Neonatal outcome after preeclampsia and HELLP syndrome: a population-based cohort study in germany. Frontiers in Pediatrics. 2020;8:579293.
14. Botet F, Figueras J, Carbonell-Estrany X, Narbona E, Group CS. The impact of clinical maternal chorioamnionitis on neurological and psychological sequelae in very-low-birth weight infants: a case-control study. 2011.
15. Claas M, Bruinse H, Van der Heide-Jalving M, Termote J, De Vries L. Changes in survival and neonatal morbidity in infants with a birth weight of 750 g or less. Neonatology. 2010;98(3):278-88.
16. Cruz-Martinez R, Tenorio V, Padilla N, Crispi F, Figueras F, Gratacos E. Risk of ultrasound-detected neonatal brain abnormalities in intrauterine growth-restricted fetuses born between 28 and 34 weeks' gestation: relationship with gestational age at birth and fetal Doppler parameters. Ultrasound in Obstetrics & Gynecology. 2015;46(4):452-9.
17. De Jesus LC, Pappas A, Shankaran S, Li L, Das A, Bell EF, et al. Outcomes of small for gestational age infants born at< 27 weeks' gestation. The Journal of pediatrics. 2013;163(1):55-60. e3.
18. Dempsey E, Chen M-F, Kokottis T, Vallerand D, Usher R. Outcome of neonates less than 30 weeks gestation with histologic chorioamnionitis. American journal of perinatology. 2005;22(03):155-9.

19. Denzler A, Burkhardt T, Natalucci G, Zimmermann R. Latency after preterm prelabor rupture of the membranes: increased risk for periventricular leukomalacia. Journal of Pregnancy. 2014;2014.
20. Ferdynus C, Quantin C, Abrahamowicz M, Platt R, Burguet A, Sagot P, et al. Can birth weight standards based on healthy populations improve the identification of small-for-gestational-age newborns at risk of adverse neonatal outcomes? Pediatrics. 2009;123(2):723-30.
21. Fernandez-Rodriguez B, de Alba C, Villalain C, Pallás CR, Galindo A, Herraiz I. Obstetric and pediatric growth charts for the detection of fetal growth restriction and neonatal adverse outcomes in preterm newborns before 34 weeks of gestation. The Journal of Maternal-Fetal & Neonatal Medicine. 2021;34(7):1112-9.
22. Gagliardi L, Bellù R, Zanini R, Dammann O. Bronchopulmonary dysplasia and brain white matter damage in the preterm infant: a complex relationship. Paediatric and perinatal epidemiology. 2009;23(6):582-90.
23. Gagliardi L, Rusconi F, Da Frè M, Mello G, Carnielli V, Di Lallo D, et al. Pregnancy disorders leading to very preterm birth influence neonatal outcomes: results of the population-based ACTION cohort study. Pediatric research. 2013;73(6):794-801.
24. Gagliardi L, Rusconi F, Bellù R, Zanini R, Network IN. Association of maternal hypertension and chorioamnionitis with preterm outcomes. Pediatrics. 2014;134(1):e154-e61.
25. Grisaru-Granovsky S, Reichman B, Lerner-Geva L, Boyko V, Hammerman C, Samueloff A, et al. Mortality and morbidity in preterm small-for-gestational-age infants: a population-based study. American journal of obstetrics and gynecology. 2012;206(2):150. e1-. e7.
26. Hendson L, Russell L, Robertson CM, Liang Y, Chen Y, Abdalla A, et al. Neonatal and neurodevelopmental outcomes of very low birth weight infants with histologic chorioamnionitis. The Journal of pediatrics. 2011;158(3):397-402.
27. Kakita H, Hussein MH, Yamada Y, Henmi H, Kato S, Kobayashi S, et al. High postnatal oxidative stress in neonatal cystic periventricular leukomalacia. Brain and Development. 2009;31(9):641-8.
28. Khazardoost S, Ghotbizadeh F, Sahebdel B, Amiri FN, Shafaat M, Akbarian-Rad Z, et al. Predictors of cranial ultrasound abnormalities in intrauterine growth-restricted fetuses born between 28 and 34 weeks of gestation: a prospective cohort study. Fetal Diagnosis and Therapy. 2019;45(4):238-47.
29. Kobayashi S, Fujimoto S, Fukuda S, Hattori A, Iwaki T, Koyama N, et al. Periventricular leukomalacia with late-onset circulatory dysfunction of premature infants: correlation with severity of magnetic resonance imaging findings and neurological outcomes. The Tohoku journal of experimental medicine. 2006;210(4):333-9.
30. Kubota H, Ohsone Y, Oka F, Sueyoshi T, Takanashi J, Kohno Y. Significance of clinical risk factors of cystic periventricular leukomalacia in infants with different birthweights. Acta paediatrica. 2001;90(3):302-8.
31. Kumazaki K, Nakayama M, Sumida Y, Ozono K, Mushiake S, Suehara N, et al. Placental features in preterm infants with periventricular leukomalacia. Pediatrics. 2002;109(4):650-5.
32. Kurahashi H, Okumura A, Kubota T, Kidokoro H, Maruyama K, Hayakawa M, et al. Increased fetal heart rate variability in periventricular leukomalacia. Brain and Development. 2016;38(2):196-203.
33. Lee DK, Kwon BS, Lee YS, Chang YP. Risk Factors for Cystic Periventricular Leukomalasia and Neurologic Outcomes According to Cranial Ultrasonography in Preterm Infants. Journal of the Korean Society of Neonatology. 2002;9(1):90-8.
34. Lee SH, Kim SH, Lee KH, You DK, Choi SJ, Hwang JH, et al. A Study on the Incidence and Risk factors of Cystic Periventricular Leukomalacia in very Low Birth Weight Infants. Journal of the Korean Society of Neonatology. 2003;10(1):61-6.
35. Lee EY, Yeom JM, Lee SH, So CH, Oh YK. Risk factors and Effect of Early Hypocarbia on the Development of Cystic Periventricular Leukomalacia in Very Low birth Weight Infants. Perinatology. 2019;30(4):229-35.

36. Lee H-S, Kim SY. Histological chorioamnionitis, antenatal steroids, and neonatal outcomes in very low birth weight infants: A nationwide study. PloS one. 2019;14(10):e0224450.
37. Leviton A, Paneth N, Susser M, Reuss ML, Allred EN, Kuban K, et al. Maternal receipt of magnesium sulfate does not seem to reduce the risk of neonatal white matter damage. Pediatrics. 1997;99(4):e2-e.
38. Maisonneuve E, Lorthe E, Torchin H, Subtil D, Marret S, Ancel P-Y, et al. 334: Impact of chorioamnionitis on neonatal neurological outcomes and mortality in premature infants born after spontaneous preterm labour or rupture of membranes. American Journal of Obstetrics & Gynecology. 2017;216(1):S202-S3.
39. Malhotra A, Yahya Z, Sasi A, Jenkin G, Ditchfield M, Polglase GR, et al. Does fetal growth restriction lead to increased brain injury as detected by neonatal cranial ultrasound in premature infants? Journal of Paediatrics and Child Health. 2015;51(11):1103-8.
40. Monier I, Ancel P-Y, Ego A, Jarreau P-H, Lebeaux C, Kaminski M, et al. Fetal and neonatal outcomes of preterm infants born before 32 weeks of gestation according to antenatal vs postnatal assessments of restricted growth. American journal of obstetrics and gynecology. 2017;216(5):516. e1-. e10.
41. Morsing E, Maršál K, Ley D. Reduced prevalence of severe intraventricular hemorrhage in very preterm infants delivered after maternal preeclampsia. Neonatology. 2018;114:205-11.
42. Murata Y, Itakura A, Matsuzawa K, Okumura A, Wakai K, Mizutani S. Possible antenatal and perinatal related factors in development of cystic periventricular leukomalacia. Brain and Development. 2005;27(1):17-21.
43. Oda N, Takeuchi K, Tanaka A, Maruo T. Obstetric risk factors associated with the development of periventricular leukomalacia in preterm infants born to mothers complicated by placenta previa. Fetal diagnosis and therapy. 2008;24(4):345-8.
44. Pappas A, Kendrick DE, Shankaran S, Stoll BJ, Bell EF, Laptook AR, et al. Chorioamnionitis and early childhood outcomes among extremely low-gestational-age neonates. JAMA pediatrics. 2014;168(2):137-47.
45. Park JM, Choi BS, Sohn IA, Seol IJ, Kim CR, Park HK, et al. Risk factors for cystic periventricular leukomalacia in very low birth weight infants. Neonatal medicine. 2014;21(3).
46. Paul DA, Kepler J, Leef KH, Siscione A, Palmer C, Stefano JL. Effect of preeclampsia on mortality, intraventricular hemorrhage, and need for mechanical ventilation in very low-birth-weight infants. American journal of perinatology. 1998;15(06):381-6.
47. Paul DA, Pearlman SA, Finkelstein MS, Stefano JL. Cranial sonography in very-low-birth-weight infants: do all infants need to be screened? Clinical pediatrics. 1999;38(9):503-9.
48. Perlman JM, Risser R, Broyles RS. Bilateral cystic periventricular leukomalacia in the premature infant: associated risk factors. Pediatrics. 1996;97(6):822-7.
49. Pladys P, Beuchee A, Wodey E, Treguier C, Lassel L, Betremieux P. Patent ductus arteriosus and cystic periventricular leucomalacia in preterm infants. Acta paediatrica. 2001;90(3):309-15.
50. Regev RH, Lusky A, Dolfin T, Litmanovitz I, Arnon S, Reichman B, et al. Excess mortality and morbidity among small-for-gestational-age premature infants: a population-based study. The Journal of pediatrics. 2003;143(2):186-91.
51. Regev RH, Arnon S, Litmanovitz I, Bauer-Rusek S, Boyko V, Lerner-Geva L, et al. Outcome of singleton preterm small for gestational age infants born to mothers with pregnancy-induced hypertension. A population-based study. The Journal of Maternal-Fetal & Neonatal Medicine. 2015;28(6):666-73.
52. Rijken M, Wit JM, Veen S. Similar growth in preterm infants with intra-or extrauterine growth restriction. A Regional Follow-Up Study at Two Years of Age in Extremely Preterm and Very Preterm Infants Leiden: Department of Paediatrics, Faculty of Medicine/Leiden University Medical Center (LUMC), Leiden University. 2007:73.
53. Rocha G, Proença E, Quintas C, Rodrigues T, Guimarães H. Chorioamnionitis and neonatal morbidity. Acta medica portuguesa. 2006;19(3):207-12.

54. Rocha G, de Lima FF, Machado AP, Guimaraes H. Preeclampsia predicts higher incidence of bronchopulmonary dysplasia. Journal of Perinatology. 2018;38(9):1165-73.
55. Rocha G, De Lima FF, Machado AP, Guimarães H, Proença E, Carvalho C, et al. Small for gestational age very preterm infants present a higher risk of developing bronchopulmonary dysplasia. Journal of neonatal-perinatal medicine. 2019;12(4):419-27.
56. Ryan E, Eves D, Menon PJ, Alnafisee S, Mooney EE, Downey P, et al. Histological chorioamnionitis is predicted by early infant C-reactive protein in preterm infants and correlates with neonatal outcomes. Acta Paediatr. 2020;109(4):720-7.
57. Salomon L, Duyme M, Rousseau A, Audibert F, Paupe A, Zupan V, et al. Periventricular leukomalacia and mode of delivery in twins under 1500 g. The Journal of Maternal-Fetal & Neonatal Medicine. 2003;13(4):224-9.
58. Shankaran S, Langer JC, Kazzi SN, Laptook AR, Walsh M, Health NIoC, et al. Cumulative index of exposure to hypocarbia and hyperoxia as risk factors for periventricular leukomalacia in low birth weight infants. Pediatrics. 2006;118(4):1654-9.
59. Shim GH, Chey MJ. Risk factors of cystic periventricular leukomalacia in preterm infants with gestational ages of less than 32 weeks according to gestational age group. Korean Journal of Perinatology. 2016;27(1):36-44.
60. Soudée S, Vuillemin L, Alberti C, Mohamed D, Becquet O, Farnoux C, et al. Fetal growth restriction is worse than extreme prematurity for the developing lung. Neonatology. 2014;106(4):304-10.
61. Spinillo A, Capuzzo E, Stronati M, Ometto A, De Santolo A, Acciano S. Obstetric risk factors for periventricular leukomalacia among preterm infants. BJOG: An International Journal of Obstetrics & Gynaecology. 1998;105(8):865-71.
62. Stewart JE, Allred EN, Collins M, Abbott J, Leviton A, Paneth N, et al. Risk of cranial ultrasound abnormalities in very-low-birth-weight infants conceived with assisted reproductive techniques. Journal of perinatology. 2002;22(1):37-45.
63. Tokumasu H, Tokumasu S, Kawakami K. Impact of pre-eclampsia in extremely premature infants: Population-based study. Pediatrics International. 2016;58(7):578-83.
64. Tyler CP, Paneth N, Allred EN, Hirtz D, Kuban K, McElrath T, et al. Brain damage in preterm newborns and maternal medication: the ELGAN Study. American journal of obstetrics and gynecology. 2012;207(3):192. e1-. e9.
65. Wagenaar N, Chau V, Groenendaal F, Kersbergen KJ, Poskitt KJ, Grunau RE, et al. Clinical risk factors for punctate white matter lesions on early magnetic resonance imaging in preterm newborns. The Journal of pediatrics. 2017;182:34-40. e1.
66. Wang L-W, Lin Y-C, Tu Y-F, Wang S-T, Huang C-C, Group TPIDCS. Isolated cystic periventricular leukomalacia differs from cystic periventricular leukomalacia with intraventricular hemorrhage in prevalence, risk factors and outcomes in preterm infants. Neonatology. 2017;111(1):86-92.
67. Weindling A, Wilkinson A, Cook J, Calvert S, Fok TF, Rochefort M. Perinatal events which precede periventricular haemorrhage and leukomalacia in the newborn. BJOG: An International Journal of Obstetrics & Gynaecology. 1985;92(12):1218-23.
68. Wiswell TE, Graziani LJ, Kornhauser MS, Stanley C, Merton DA, McKee L, et al. Effects of hypocarbia on the development of cystic periventricular leukomalacia in premature infants treated with high-frequency jet ventilation. Pediatrics. 1996;98(5):918-24.
69. Wu P-M, Wu C-Y, Li C-I, Huang C-C, Tu Y-F. Association of cystic periventricular leukomalacia and postnatal epilepsy in very preterm infants. Neonatology. 2023;120(4):500-7.
70. Yamakawa T, Itabashi K, Kusuda S, Japan NRNo. Mortality and morbidity risks vary with birth weight standard deviation score in growth restricted extremely preterm infants. Early human development. 2016;92:7-11.

71. Zeitlin J, El Ayoubi M, Jarreau PH, Draper ES, Blondel B, Künzel W, et al. Impact of fetal growth restriction on mortality and morbidity in a very preterm birth cohort. J Pediatr. 2010;157(5):733-9.e1.
